# Deep Mutational Scan of the Highly Conserved Influenza A Virus M1 Matrix Protein Reveals Substantial Intrinsic Mutational Tolerance

**DOI:** 10.1128/JVI.00161-19

**Published:** 2019-06-14

**Authors:** Nancy Hom, Lauren Gentles, Jesse D. Bloom, Kelly K. Lee

**Affiliations:** a Department of Medicinal Chemistry, University of Washington, Seattle, Washington, USA; b Department of Microbiology, University of Washington, Seattle, Washington, USA; c Division of Basic Sciences, Fred Hutchinson Cancer Research Center, Seattle, Washington, USA; d Howard Hughes Medical Institute, Chevy Chase, Maryland, USA; University of Kentucky College of Medicine

**Keywords:** M1 matrix, codon library, deep mutational scan, influenza A virus, mutational tolerance, selection, viral evolution

## Abstract

Influenza A virus matrix protein M1 is involved in multiple stages of the viral infectious cycle. Despite its functional importance, our present understanding of this essential viral protein is limited. The roles of a small subset of specific amino acids have been reported, but a more comprehensive understanding of the relationship between M1 sequence, structure, and virus fitness remains elusive. In this study, we used deep mutational scanning to measure the effect of every amino acid substitution in M1 on viral replication in cell culture. The map of amino acid mutational tolerance we have generated allows us to identify sites that are functionally constrained in cell culture as well as sites that are less constrained. Several sites that exhibit low tolerance to mutation have been found to be critical for M1 function and production of viable virions. Surprisingly, significant portions of the M1 sequence, especially in the C-terminal domain, whose structure is undetermined, were found to be highly tolerant of amino acid variation, despite having extremely low levels of sequence diversity among natural influenza virus strains. This unexpected discrepancy indicates that not all sites in M1 that exhibit high sequence conservation in nature are under strong constraint during selection for viral replication in cell culture.

**IMPORTANCE** The M1 matrix protein is critical for many stages of the influenza virus infection cycle. Currently, we have an incomplete understanding of this highly conserved protein’s function and structure. Key regions of M1, particularly in the C terminus of the protein, remain poorly characterized. In this study, we used deep mutational scanning to determine the extent of M1’s tolerance to mutation. Surprisingly, nearly two-thirds of the M1 sequence exhibits a high tolerance for substitutions, contrary to the extremely low sequence diversity observed across naturally occurring M1 isolates. Sites with low mutational tolerance were also identified, suggesting that they likely play critical functional roles and are under selective pressure. These results reveal the intrinsic mutational tolerance throughout M1 and shape future inquiries probing the functions of this essential influenza A virus protein.

## Abstract (precis)

The M1 matrix protein is critical for many stages of the influenza virus infection cycle. Currently, we have an incomplete understanding of this highly conserved protein’s function and structure. Key regions of M1, particularly in the C terminus of the protein, remain poorly characterized. In this study, we used deep mutational scanning to determine the extent of M1’s tolerance to mutation. Surprisingly, nearly two-thirds of the M1 sequence exhibits a high tolerance for substitutions, contrary to the extremely low sequence diversity observed across naturally occurring M1 isolates. Sites with low mutational tolerance were also identified, suggesting that they likely play critical functional roles and are under selective pressure. These results reveal the intrinsic mutational tolerance throughout M1 and shape future inquiries probing the functions of this essential influenza A virus protein.

## INTRODUCTION

The rapid evolution of the surface glycoproteins hemagglutinin (HA) and neuraminidase (NA) on influenza virus has been well established (1–3). Exposed residues on these two proteins evolve rapidly because they are under strong selective pressures from the host immune system and because these proteins have a high degree of mutational tolerance that allows for antigenic variation while preserving functionally and structurally critical sites that are more highly conserved (4–6). The internal influenza A virus (IAV) proteins evolve more slowly, likely due in part to the fact that they are under less intense immune selection than surface proteins and, for some at least, because they may be less inherently tolerant of mutations (2, 7–9). The specific reasons for the slower evolution of these components are starting to be dissected through studies that can decouple a protein’s intrinsic tolerance to variation from the more complex selective pressures present in natural influenza virus infections.

The M1 matrix protein is a 252-residue structural protein in influenza virus that oligomerizes into an endoskeleton-like coat beneath the viral lipid bilayer. It plays a critical role in recruiting other viral components during budding assembly and interacts with viral ribonucleoproteins (vRNPs) as well as the cytoplasmic tails of HA and NA surface proteins (10–15). M1 is encoded by the M gene, which also encodes the M2 proton channel as a splice variant. In human seasonal influenza viruses, the M gene has been reported to evolve 5- to 10-fold more slowly than the HA gene, although there is a difference in evolutionary rates between the coding regions for M1 and M2, with M2 evolving somewhat more rapidly than M1 (16, 17). Indeed, two recent studies indicate that M1 is one of the slowest-evolving proteins encoded by the influenza virus genome (16, 18). While there are differences in M gene evolutionary rates between viruses infecting different host species, IAV strains sampled globally in humans and across a range of other host species exhibit over 95% amino acid sequence identity for the M1 protein (16, 18–20).

The relatively low apparent variation of M1 sequences may in part be due to a lack of intensive M1 sequence sampling, or it may be due to inherent constraints on M1 evolvability. Indeed, the reasons for the high degree of conservation of M1 observed in nature are not immediately clear, though it appears that several factors likely influence its evolution. Like other internal proteins, M1 is sequestered from humoral immune pressure and hence does not experience pressure to adopt mutations to escape antibody selection. Portions of M1 do appear to be targets for CD8 and CD4 T cells (21–23). A particularly common M1 CD8 T-cell epitope, for example, overlaps a suspected critical nuclear transport sequence (21, 22). However, in general, it has been difficult to detect immune selection pressure on M1 that would contribute to diversification of the protein’s sequence (7).

Another key factor that likely underlies its observed conservation is that M1 plays several critical functions in the influenza A virus infectious cycle and participates in interactions with multiple viral and host proteins. Such multifaceted functional roles impose significant constraints on M1’s ability to evolve. In addition to its role in recruiting multiple viral components at assembly budding sites, M1 plays pivotal roles in determining particle morphology, promoting an orderly sequence to membrane fusion, and trafficking vRNP following cell entry (24–32). Additionally, M1 interacts with a variety of host proteins, which phosphorylate and SUMOylate several sites in M1. Such modifications are necessary for M1 to carry out a number of its functions (33–40).

In this study, we sought to determine whether the high degree of evolutionary conservation observed for influenza virus M1 is due to inherent mutational intolerance or whether the protein may be more permissive of residue substitutions than the highly conserved natural sequences would indicate. To test this, we performed a deep mutational scan (DMS) of M1 matrix protein (41, 42). We selected mutant libraries containing all possible single amino acid mutations to MI for viral replication in mammalian cells. Deep sequencing of the starting DNA and functional virus libraries was used to measure how well tolerated each amino acid is at each site in M1. Surprisingly, M1 was found to possess significant regions of mutational tolerance that contrasted dramatically with the limited sequence variation observed in nature. These studies lay the foundation for understanding how M1 function is encoded in the amino acid sequence and how those functional requirements constrain the gene’s evolution.

## RESULTS

### M1 codon mutant library thoroughly samples all codon mutations.

**FIG 1:**
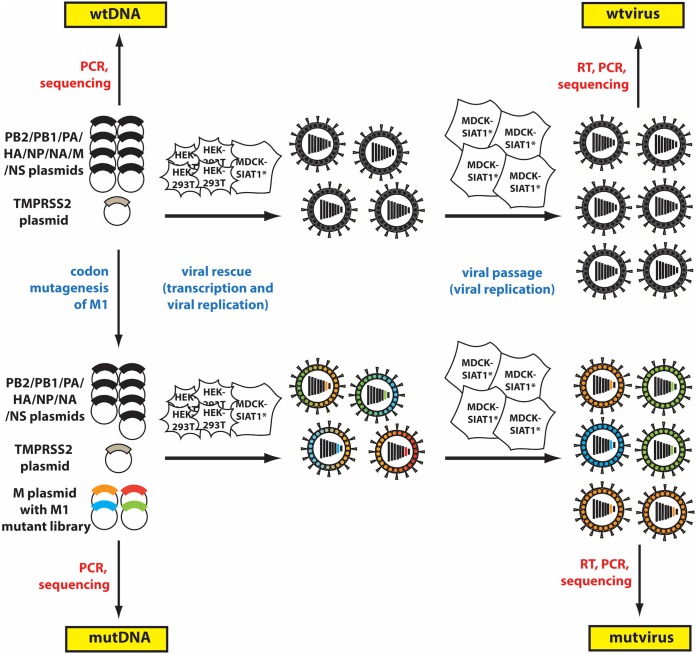
Scheme for deep mutational scan (DMS) of the M1 gene in X31 virus. The procedure is initiated by carrying out transfection of reverse genetic plasmids into a HEK293T and MDCK-SIAT1-TMPRSS2 cell coculture and then passaging virus to select for functional viral mutants that are replication competent. Samples for deep sequencing and analysis with dms_tools2 are highlighted in yellow. Samples of wtDNA and mutDNA, representing prereplication selection conditions, were sequenced directly from the plasmid library, while wtvirus and mutvirus samples, representing postreplication selection conditions, were reverse transcribed from viral RNA before sequencing.

Our strategy for the deep mutational scan scheme of M1 is outlined in Fig. 1. We mutagenized M1 from the X31 H3N2 influenza A virus strain M segment at the codon level to create triplicate DNA plasmid mutant codon libraries that comprehensively incorporate all possible ∼16,000 single-site codon mutations. To do so, we used the PCR strategies from Bloom, 2014, implementing NNN codon primers that overlap each codon site across the 252-amino-acid-long M1 gene and inserting the mutagenized amplicons into a pHH21 reverse genetics plasmid (43). While amino acid sites 1 to 9 and 239 to 252 of the M1 sequence overlap the coding region for M2 amino acids 1 to 24 (44), in this study, only the regions of the M gene segment corresponding to in-frame codons for M1 were subjected to mutation; nonoverlapping portions encoding M2 were not mutated. Three replicates of the M1 plasmid mutant codon library were created.

**FIG 2:**
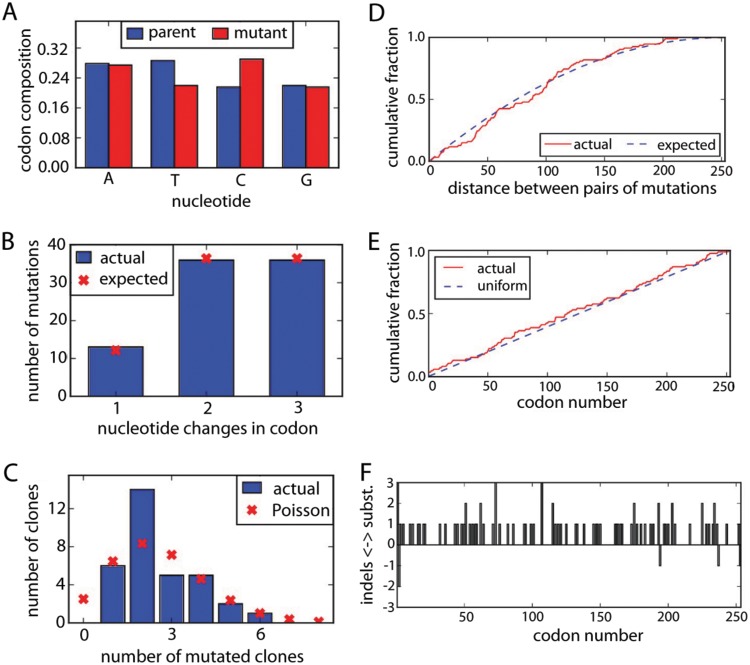
Initial assessment of codon libraries by Sanger sequencing of a combined 33 transformant clones of the three mutDNA replicates. (A) The nucleotide composition of the mutant libraries is roughly uniform. (B) The mutant library exhibits 1-, 2-, and 3-nucleotide changes. (C) An average of 2.6 codon mutations per clone, roughly following a Poisson distribution, was observed. (D) There is no tendency for mutations to cluster, as reflected by the pairwise distance between mutations. (E) The mutations are distributed evenly across the M1 gene. (F) The rate of insertion and deletion mutations is 0.2 per clone. The software to generate this figure is found at https://github.com/jbloomlab/SangerMutantLibraryAnalysis.

In order to assess whether all codon mutations were thoroughly represented in our M1 library triplicates, we initially Sanger sequenced 33 transformant clones from the M1 plasmid libraries and observed an average of 2.6 codon mutations per clone with a Poisson distribution around the mean and a low level of insertions and deletions (Fig. 2). For each final DNA library replicate, we aimed to attain a diversity of at least 2.5 million unique clones, which theoretically covers each single codon mutation over 150 times.

To ensure good sampling of the 15,876 individual codon mutations in the final M1 plasmid mutant codon library, we determined the number of times each codon mutation was observed by deep sequencing the DNA plasmid libraries, which yields mutated DNA (mutDNA) samples (Fig. 1). We observed that for each replicate, in the mutDNA samples (before selection), ∼90% of codon mutations have a high count (e.g., 15+) (Fig. S1A). We thus concluded that the codon diversity in the plasmid libraries was thorough and comprehensive.

### Passaging of virus leads to purifying selection against deleterious mutations.

The M1 plasmid mutant codon library and seven other plasmids containing the remaining influenza virus gene segments along with a plasmid encoding the TMPRSS2 protease to facilitate HA cleavage maturation were used for reverse genetics transfection to rescue virus. The transfection of the plasmids into a coculture of HEK293T and MDCK-SIAT1-TMPRSS2 cells (45) was followed by passaging selection of the rescued virus at a low multiplicity of infection (MOI) in a monoculture of MDCK-SIAT1-TMPRSS2 cells to ensure a stringent genotype-phenotype link in the final virus pool. This resulting final virus population was deep sequenced to identify the frequency of codon mutations present in virus that was able to successfully replicate in cell culture, yielding postselection mutant virus (mutvirus) samples (Fig. 1). The entire experimental pipeline was completed in biological triplicate.

**FIG 3:**
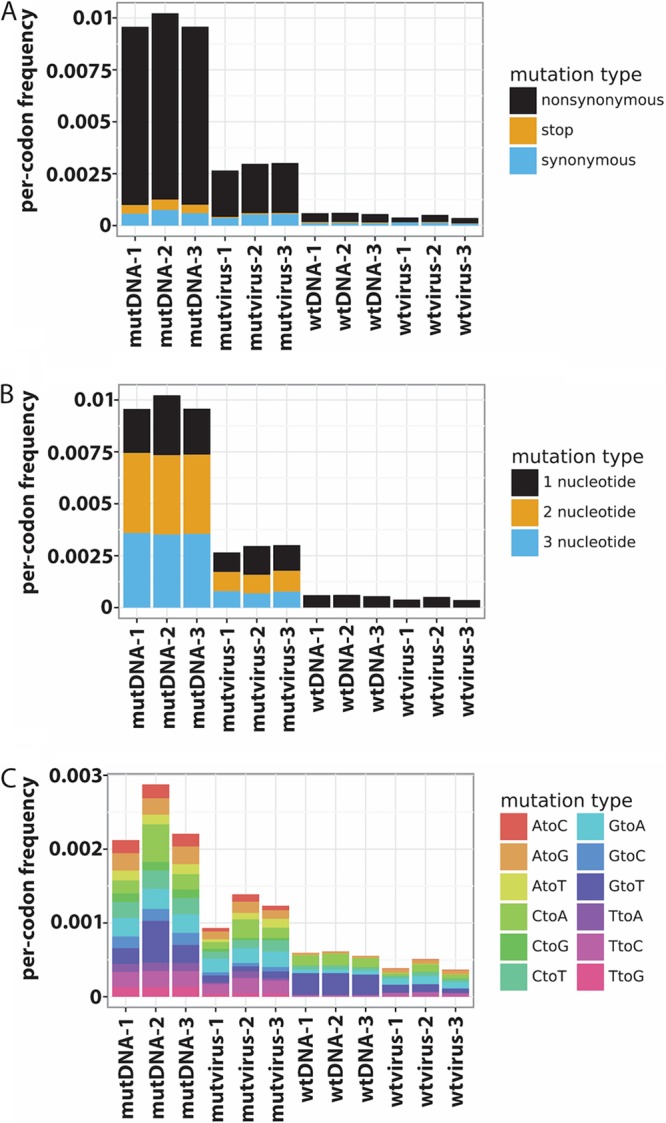
Analysis of codon mutation types sampled in each of the three replicates. (A) Most of the mutations in mutDNA samples are nonsynonymous. After replication selection, deleterious mutations (mostly coding for nonsynonymous and stop codons) are purged, as observed in mutvirus samples. Mutations in wtDNA and wtvirus control samples are very low. (B) As expected from codon mutation primers, there is a distribution of 1-, 2-, and 3-nucleotide mutations in mutDNA and mutvirus samples. An overall lower frequency of mutations is observed in mutvirus (postselection) compared to mutDNA (preselection) due to purifying selection. Most mutations in wtDNA and wtvirus samples are single nucleotide mutations, which may have arisen from PCR transcription, barcoding, and sequencing errors. (C) Assessment of the frequency of nucleotide mutations by type among only single-nucleotide codon mutants. After mutDNA samples undergo purifying selection, the frequency of mutation decreases in mutvirus samples. As expected, the mutation frequency in wtDNA and that in the wtvirus samples are lowest. The wtDNA samples show a small amount of oxidative damage as indicated by a slightly increased rate of G**-**to**-**T mutations.

By comparing mutation frequencies before passaging (mutDNA) and after passaging (mutvirus), we could determine the extent of purifying selection that occurred during replication in cell culture. We observed, on average, a 3.4-fold decrease in codon mutation frequency in the mutvirus samples, indicating strong purifying selection and purging of deleterious mutations from the mutDNA library (Fig. 3A). The reduction in mutation frequency is mostly in stop codon and nonsynonymous codon frequencies in mutvirus compared to mutDNA (Fig. 3A). It is expected that stop codons and nonsynonymous codon mutations in M1 are more deleterious than synonymous mutations during viral replication.

In order to confirm that the changes in codon mutation frequency we observed were not due to spurious noise introduced by the reverse transcription or other intermediate steps, we examined the codon mutation frequency of wild-type DNA and virus (wtDNA and wtvirus) control samples. In both the preselection wtDNA and postselection wtvirus control samples, a low frequency of codon mutations overall was observed, most of which are single nucleotide mutations (Fig. 3B). Single nucleotide codon mutations may arise from errors during the PCR amplification, reverse transcription, barcoding, and deep sequencing steps or, in the case of wtvirus, during error-prone viral replication. However, we observed similarly low frequencies of codon mutations in the wtDNA and the wtvirus samples (Fig. 3), indicating that reverse transcription of viral RNA to make sequencing amplicons introduced few, if any, mutations into the samples of rescued virus. The preselection mutDNA and the postselection mutvirus samples had a higher frequency of mutations than wtDNA and wtvirus, as anticipated (Fig. 3 and Fig. S1). Among the codon mutations in the mutDNA and mutvirus samples, we observed a distribution of single-, double-, and triple-nucleotide codon mutations, which was expected based upon the primer designs for codon library generation (Fig. 3B). Furthermore, we checked for biases that arose during the generation of the mutant codon libraries by looking at the frequencies of each type of single nucleotide change that occurred (Fig. 3C). We observed a reasonable distribution of single nucleotide changes, with about twice as many G-to-T mutations in the mutDNA-2 library relative to mutDNA-1 or mutDNA-3 libraries, which may be an indication of slight oxidative damage during library preparation for this replicate. Overall, by comparison with the wtDNA and wtvirus controls, we determined that passaging of the virus rescued from the mutDNA libraries led to enrichment of favorable mutations and depletion of deleterious mutations in the mutvirus samples.

**FIG 4:**
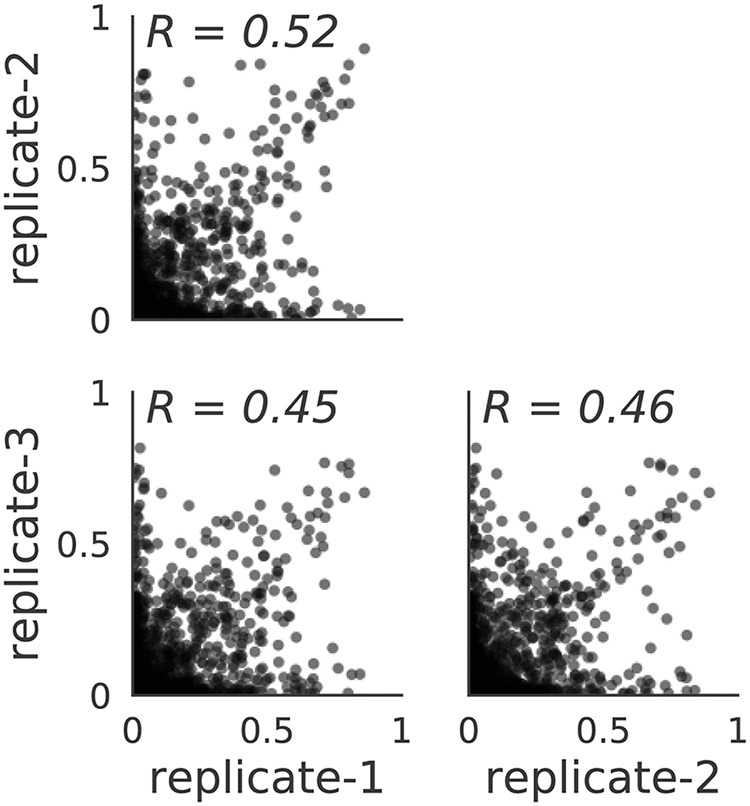
Correlation among the amino acid preferences from three replicates. The correlation is substantial, but there is an observable degree of variation between replicates. This indicates that there are differences in the genotypes of the predominate rescued virus in each replicate. By combining the data, there is more complete sampling of the inferred M1 amino acid preferences.

To quantify the extent of error between our three biological replicates, we examined the correlation between the independent experiments. We observed a good positive correlation between our three replicates, with *R* values between 0.45 and 0.52 (Fig. 4). The variation observed between replicates is attributable primarily to the ∼50% sampling of possible amino acids with 5 counts or less after selection, as we observed in each independent mutvirus pool (Fig. S1A). The selection for a specific amino acid in each replicate may differ, indicating that separately, each independent replicate may not completely sample all M1 variants (bottlenecking). However, by combining all replicates, we can improve the accuracies and completeness of our amino acid selection and strengthen the total diversity represented in the mutvirus pool of selected viruses.

### Estimation of amino acid preference and number of effective amino acids tolerated (*N*_eff_) at every M1 site.

**FIG 5:**
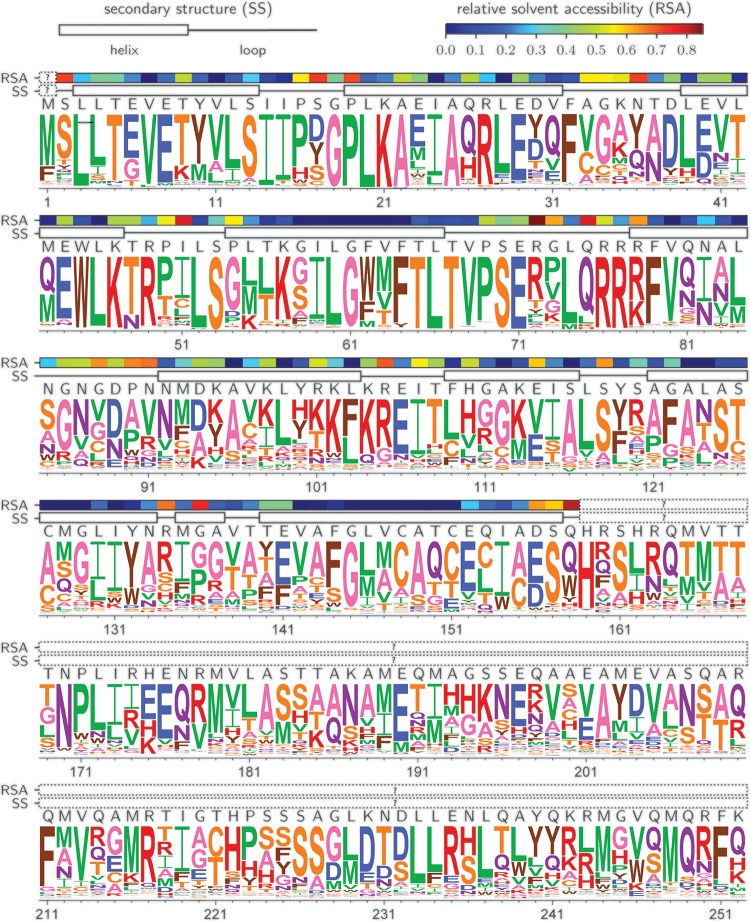
Logo plot representing the amino acid preferences at each residue site inferred from the combined data of all three replicates. Heights of the amino acid letter are proportional to the preference of that amino acid at that site. The plot is scaled by a stringency factor, β, which determines how well the selection in our experimental data matches the stringency of selection in nature (46). The wild-type sequence is indicated above the logo plot. For the portion of the M1 that has a crystal structure, residues 2 to 158 (PDB code 1EA3), the secondary structure and the relative solvent accessibility (RSA) are represented as overlay bars. The secondary structure and absolute solvent accessibilities for this plot were calculated using DSSP (75), and then the absolute solvent accessibilities were normalized to RSAs using the maximum solvent accessibilities (76).

Amino acid types at each position that are favored during viral replication in the selection passage result in high representation in the resulting virus pool and a high apparent preference as previously defined (46), while those that are disfavored and purged from the virus pool have low representation and low preference. We quantified the amino acid preferences at every residue site using the dms_tools2 package (46) and generated an amino acid preference logo plot to summarize the results (Fig. 5). The preferences in Fig. 5 show the average values across the combined experimental replicates and indicate the average effect of each amino acid from all sequence contexts in which it is sampled. For this plot, we used phydms software (47) to scale our preferences by a stringency parameter (β), calculated by comparing our results with a phylogenic tree of M1 sequences from 1918 to 2013, as found in the publication by Machkovech et al. (7). A β value greater than 1 indicates that selection in nature prefers the same amino acid residues as our experimentally informed model but with greater stringency. A β value of less than 1 indicates that our experimentally determined preferences differ from selection seen in natural virus evolution. Our DMS preference data resulted in a β value of 1.96, suggesting that amino acids that are preferred in our deep mutational scanning selections are also preferred, but with considerably more stringency, in nature.

**FIG 6:**
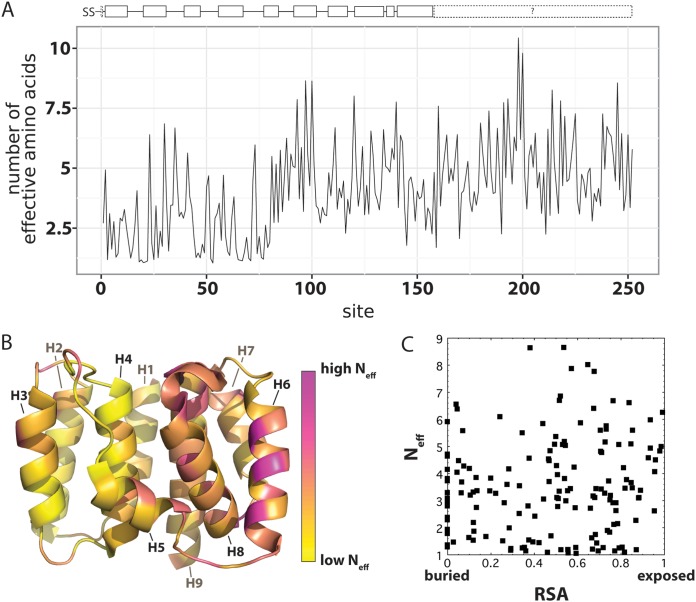
Mutational tolerance per residue site represented as *N*_eff_, the number of effective amino acids per position. (A) *N*_eff_ variation as a function of primary sequence; (B) *N*_eff_ plotted as a heat map onto the known truncated crystal structure of residues 2 to 158 (PDB code 1EA3). (C) *N*_eff_ does not exhibit a strong correlation with a residue’s relative solvent accessibility (RSA), with an *R* of only 0.10. This likely reflects the missing contacts with other viral components, including membrane, that numerous residues make in M1’s native context in virions.

The distribution of mutational tolerance across the M1 protein revealed a notable domain-specific profile (Fig. 6). Sites with a preference dominated by a single amino acid (low mutational tolerance), which mostly matched the naturally occurring wild-type amino acid, were highly abundant across the first 80 residues of M1. In contrast, in the C-terminal two-thirds of M1, sites with low mutational tolerance were more sparsely distributed and rare, while numerous sites were observed to exhibit a high tolerance for a variety of amino acids. Our data can also be depicted as the number of effective amino acids per position (*N*_eff_). Sites with a low tolerance for mutations have a low number of effective amino acids, while sites with a high tolerance for mutations are sites with a high number of effective amino acids. By mapping the values along the M1 protein, it is evident there is a trend that the N-terminal third in general has a lower *N*_eff_ than the C-terminal two-thirds of M1 (Fig. 6A). We mapped the *N*_eff_ values onto the truncated M1 crystal structure (PDB code 1EA3), which only covers residues 2 to 158, including helices 1 to 9 (48) (Fig. 6B). Positions with a low *N*_eff_ clearly segregate to one side of the M1 subunit monomer, spanning helices 1 to 4. Helix 6 (residues 92 to 103) appears to be the most permissive to mutation, with a generally high *N*_eff_ observed (Fig. 6). The C-terminal third is not available in any crystal structure of M1. These C-terminal residues are, on average, relatively tolerant of mutations, with high *N*_eff_ values (Fig. 5 and 6), which may reflect a recently proposed disordered configuration for the C-terminal domain of M1 (49).

Additionally, in Fig. 5, above the logo plot, we show the relative solvent accessibility (RSA) calculated for the portion of M1 that has a crystal structure available (PDB code 1EA3), since residue burial often correlates with intolerance to mutation. In order to draw a comparison between the DMS data with available structural information, we examined whether a correlation between RSA and the *N*_eff_ exists (Fig. 6C). Based on previous reports (50, 51), one expects that greater burial of residues (or those with low RSA) would impose greater constraints on a residue’s tolerance for mutation, leading to a low *N*_eff_, while surface residues (with high RSA) would be under less constraint, leading to a high *N*_eff_. However, in our data for M1, we did not observe a clear correlation between *N*_eff_ and RSA. Surprisingly, we observed that highly exposed residues with high RSA exhibit a range of *N*_eff_ values, including numerous residues that have very low mutational tolerance (low *N*_eff_) (Fig. 6C). We note that the RSA calculated from the truncated monomer crystal structure, however, does not account for the many interactions M1 has with viral membrane, RNP, and other M1 subunits. These structural and functional interactions very likely also constrain these residues’ ability to tolerate mutations.

### Comparison to an error-prone PCR analysis of M1 shows a modest correlation.

In a previous study, Wu and coworkers used error-prone PCR to investigate IAV M1 epistatic mutations (52). In comparing our comprehensive DMS analysis with their resulting M1 genetic library, we found that the two data sets exhibited a modest correlation, indicated by the relatively low to moderate *R* of 0.31 (Fig. S2). The differences in findings likely can be attributed to experimental noise and differences in methodology. Wu and coworkers used the A/WSN/1933 (H1N1) strain passaged with A549 cells, while we used the X31 strain (Aichi/68 H3N2 external proteins with an H1N1 PR8 background, including M1), passaged with MDCK-SIAT1 cells. Additionally, we cannot rule out the possibility of noise from sources such as sequencing errors or population bottlenecks contributing to the differences observed between the data sets from Wu et al. and our DMS study. For instance, as described above, replicates of our experiments only had a correlation of 0.45 to 0.52 with each other, and the replicates in the experiments of Wu and coworkers had similarly low correlations. Furthermore, as a result of using only single nucleotide mutations, Wu and coworkers reached only ∼20% of the diversity of our library, which had 1-, 2-, and 3-nucleotide (nt) codon changes to M1. We note that Wu et al. observed that the N-terminal third of the protein had very low tolerance to mutations, which agrees with our results (Fig. 5 and 6). In their study, the most mutable, least “essential” sites of M1 were concentrated in the middle portion of the protein, ∼residues 80 to 170, a region where a relatively high degree of mutational tolerance was observed by our DMS study (Fig. 5 and 6). Interestingly, Wu et al. observed similar mutability between the N- and C-terminal domains, while our data suggest that, on average, the C-terminal domain exhibits significantly greater mutational tolerance than the N-terminal domain.

## DISCUSSION

The M1 matrix protein plays a pivotal role in several stages of the influenza virus infectious cycle. Despite its importance, our understanding of this protein remains relatively fragmented. This incomplete understanding is due in part to the difficulty of analyzing M1 in isolation and to the lack of a complete structure for M1 in the context of intact virions as well as in complex with its numerous interaction partners from both host cells and virus. The high degree of sequence conservation across influenza virus subtypes isolated in nature further obscures straightforward identification of which residues may be functionally critical. In this study, we used DMS to characterize the intrinsic tolerance to mutation of every residue in the M1 protein. This comprehensive, protein-wide map of mutational tolerance and amino acid preference revealed surprisingly high levels of mutational tolerance across substantial regions of the protein. Our analysis also helps to distinguish M1 sites that are under selection and play critical functional roles, reflected by stringent amino acid preference at those sites. The M1 mutant library forms the basis for future studies in which a more targeted selection is applied in order to identify sites linked with those selective pressures.

### Structural influences on IAV M1 mutational tolerance.

Previous studies have suggested that the M1 protein can be segmented into N-terminal (N), linker (L), and middle (M) domains as well as the C-terminal subdomains (53). The N, L, and M regions have been resolved in several crystal structures (48, 54–56) and appear to form a brick-like domain composed of 9 α-helices. The N subdomain spanning the first third (residues ∼1 to 66) has been reported to play a role in M1 oligomerization, the linker domain (residues 67 to 86) has been implicated in M1 association with membranes, and the M domain (residues 88 to 158) has been reported to mediate interactions between M1 subunits as well as interactions with RNP (53). We can use DMS to examine the mutational tolerance of each of these domains.

The portions of IAV M1 with the overall highest mutational tolerance were observed within the C-terminal third of the protein (residues 159 to 252), which has yet to be fully resolved in any high-resolution structures. Studies have indicated that the full-length M1 subunit, including this domain, is necessary for efficient matrix layer assembly and budding (53). It is possible that the relatively high tolerance to mutation is consistent with reports that the C-terminal domain exhibits characteristics of an intrinsically disordered domain (49). Solution small-angle X-ray scattering (SAXS) of full-length, nonassembled IAV M1 suggested that the C-terminal domain may be unstructured or extended in the monomeric subunit, at least under acidic pH conditions. An absence of residue packing interactions would plausibly reduce constraints on amino acid preferences, perhaps leading to a greater degree of mutational tolerance. In contrast, a recent structure of a full-length M1 protein from a distantly related orthomyxovirus, infectious salmon anemia virus (ISAV), revealed the organization of the “N-domain” (corresponding to the NLM domain, residues 1 to 158, observed for available influenza A virus M1 matrix structure) as well as an ordered, helical “C-domain” (corresponding to the analogous C-terminal domain in influenza virus) (57). The full-length ISAV subunits in the crystal were polymerized into 2-dimensional arrays that were proposed to recapitulate how M1 is assembled inside orthomyxovirus virions. ISAV M1 N-domains formed the primary contact between subunits, while the C-domains appeared to play less of a dominant role in mediating interactions in the crystalline array. We note that ISAV M1 is considerably smaller than influenza A virus M1 protein (196 versus 252 residues); hence, it is not certain whether the proteins will exhibit similar overall subunit structures with ordered C-terminal domains or assemble into similar arrays involving similar intersubunit contacts.

### Impact of genomic factors on M segment evolvability.

In addition to protein structural requirements, RNA packaging signals that may impose constraints on mutational variability exist within the 5′ and 3′ coding regions of all IAV gene segments. Likewise, other functional RNA motifs, such as secondary structures and regulatory *cis*-acting signals (e.g., splice donor and acceptor sequences), for segments like M and NS that encode multiple protein products need to be considered (58). These features, essential for viral replication, likely also impose selective constraints upon the M gene. Hutchinson and coworkers explored nucleotide sequence conservation in relation to *cis*-acting signals within the N terminus of M1; when synonymous mutations were made to highly conserved codon sites 7, 8, 9, 18, 19, and 20 of M1, they observed a 10- to 100-fold reduction in viral tiers (59). Gog and coworkers also found evidence for selection of M1 codons in this region by examining a panel of IAV sequences (60). These types of functions at the level of the RNA genome likely contribute to constraining mutational variability of the N-terminal region of the M gene.

Another important contributing factor that warrants consideration is the overlap in coding regions shared by M1 and M2, the 97-residue membrane-embedded influenza virus ion channel (44). The first area of overlap spans the in-frame codons for the first 9 residues of both proteins. These M1 and M2 residues exhibit extremely low tolerance for mutations, with the most preferred amino acids in the DMS analysis matching the highly conserved, wild-type consensus sequences (61, 62). In the second area of coding overlap, a frameshift occurs between the C-terminal residues 239 to 252 of M1 and residues 10 to 24 in M2 (44). The M1 DMS data for these residues exhibit various degrees of high and low *N*_eff_ values. In order to understand this overlapping area better, we examined specific residues of M2 with reported function to see how M2 may influence M1’s amino acid preference. One notable feature of M2 residues 10 to 24 involves the two cysteines at positions 17 and 19, which are highly conserved and participate in disulfide bridges to hold the M2 tetramer together (61, 63). In the case of C19 of M2, this residue overlaps with M248 and Q249 in M1, which are highly preferred in our mutational tolerance map, with low tolerance for mutations and a low *N*_eff_ (Fig. 5 and 6). This suggests a strong constraint involving both M1 and M2 proteins at this site. In the case of C17 of M2, the residue overlaps with M1 V246 and Q247, sites we observed to be fairly mutationally tolerant (Fig. 5 and 6). Research by Kwon and Hong demonstrated that M2 variants with either of the cysteines at positions 17 and 19 mutated to a serine and then embedded in a bilayer led to nuclear magnetic resonance (NMR) signals similar to those of a wild-type protein also bilayer embedded (61). This suggests that one cysteine at a time may suffice for proper function of M2, with C19 perhaps playing a more critical role for M2. A more complete assessment of the intrinsic mutational tolerance of M2 may shed further light on its functional and sequence constraints as well as its genetic linkage to M1.

### Constraints on mutability are apparent at key functional sites.

While there seem to be relatively high levels of mutational tolerance across large regions of M1, our data reveal that many other sites also exhibit low tolerance for mutations, including in the generally permissive C-terminal domain. These sites are likely subject to a high degree of functional constraint. Some of these constrained sites correspond or are proximal to previously reported amino acids that play specific roles in M1 function (Table 1). For example, the low mutational tolerance of this N-terminal third may reflect the reported role of this subdomain to participate in M1-M1 assembly and oligomerization (53). Portions of the L linker region extending to the highly conserved _76_RRR_78_ motif are also highly constrained. This motif has been implicated in electrostatic interactions with the negatively charged inner leaflet of the viral membrane (64, 65). Kerviel and coworkers found this arginine triplet, which spans a loop and helix 5, to be critical for M1 incorporation into virus particles (66). We find that arginines at positions 76 and 77 are essential, while a relatively conservative lysine substitution is permissible in place of R78. We further note that this triplet is buttressed by highly preferred hydrophobic, likely membrane-interacting residues L74, F79, and V80 (67).

**TABLE 1:** Comparison of DMS data with previously characterized, functionally important M1 residues

Site	Existing knowledge	DMS inferred amino acid preferences from this study
S2/T5/T9/Y10	Participates in a negatively charged patch when phosphorylated (35).	The wild-type amino acids are the most preferred at these 4 sites.
V41	Mutation to A confers filamentous phenotype and higher transmissibility (77).	In our data with spherical X31 strain, V is most preferred and is over 47 times more preferred than A.
59-ILGFVFTLTV-68	Nuclear export signal (78). Also, this sequence overlaps with a known T-cell epitope from residues 58–66 (22).	Eight of 10 wild-type residues are the most preferred. At positions 62 and 63, F and V are the second most preferred residues.
I59	Site 59 strongly influences viral rescues. An A at this site cannot be tolerated (79).	At site 59, I is the most preferred, and it is over 918 times more preferred than A.
76-RRR-78	Triple R is important for binding membrane (66).	The wild-type R is most preferred at sites 76, 77, and 78.
101-RKLKR-105	Nuclear localization signal (30, 78).	R at 101 is third most preferred (K is most preferred; T is second); K at 102 is most preferred; L at 103 is second most preferred (F is first); K at 104 is most preferred; R at 105 is most preferred.^*a*^
Y132	Phosphorylation needed for binding to nuclear import factor importin α1 (36).	Y is the most preferred amino acid.
C148-C151-H159-H162	Zn^2+^ interaction (68).	The most preferred amino acids at sites 148, 151 and 159 match those of the wild type. At site 162, H is 3rd most preferred.^*a*^
K242	Sumoylation is important for export (33).	K is the second most preferred amino acid.^*a*^

a Some residue(s) in this sequence/site may have a higher mutational tolerance than indicated by the natural evolution of the M1 sequence.

In addition, Ye and coworkers found that residues _101_RKLKR_105_ are necessary for M1 to be transported to the nucleus of the cell (30). In crystal structure, residues _101_RKLKR_105_ form an exposed loop that is between helix 6 and helix 7, which is on the surface of the N-terminal structure and may be useful in interacting with host factors and viral RNP. From our analysis, it appears that a conservative lysine substitution for R101 and a conservative phenylalanine substitution for L103 are viable substitutes. However, for the other three residues in this motif, the natural amino acid is highly preferred. Such residues with low mutational tolerance likely reflect the essential functional role this motif plays in the influenza virus infectious cycle.

As an additional example of such an alignment of functional constraint and low tolerance to mutation, it has been reported that residues C148, C151, H159, and H162 of M1 may be important for M1’s pH sensing during endocytic uncoating (68). Residues C148 and C151 are in helix 9 of the known, truncated M1 crystal structure, while H159 and H162 were not resolved (49, 55, 56, 68, 69). Based on our DMS analysis, these sites exhibit high preferences for the natural wild-type amino acid, indicating that they indeed play critical functional roles in M1. Numerous other sites likely also are under functional selection (summarized in Table 1), leading to a limited tolerance to mutation, but most have yet to be examined in functional studies. We note that a number of residue positions in the otherwise mutationally tolerant C-domain exhibit a high degree of preference for specific amino acid types. While the majority of these align with the naturally observed amino acid at each given position, some residues in our study, such as site 211, showed notable deviations in the preferred amino acid (phenylalinine) relative to the naturally occurring amino acid type (glutamine). Future targeted mutation studies can probe the functional role of these mutationally constrained sites.

### Comparison of natural sequence variation and DMS data.

In addition to observing low tolerance at functionally critical sites that are highly conserved in natural sequences, we also observed large regions in which a low variation (high conservation) in nature stood in stark contrast to high degree of mutational tolerance in our DMS data. The reasons why specific amino acids at numerous mutationally tolerant sites appear to be fixed in the natural population is not clear based upon available information from this study. It is possible that natural evolution of M1 has simply not yet sampled all possible amino acids at these sites. Alternatively, the *in vivo* host environment and requirements for infection as well as interhost transmission may impose significant selective constraints on naturally circulating influenza virus strains that are not present in tissue culture propagation of influenza virus. Indeed, among natural infections strains, Furuse and coworkers found that more than 60% of the codon sites of M1 across many influenza virus hosts are under significant negative selection, leading to retention of wild-type amino acids, perhaps due to functional constraints of the protein or interactions with host factors (16).

By generating a comprehensive map of mutational tolerance across the M1 gene, we have demonstrated that the multifunctional M1 protein is considerably more permissive of residue substitutions than the naturally occurring sequences would indicate. This points to the possibility that selective pressures in host organisms or during transmission may impose further constraints upon M1’s evolvability. Our DMS map also identifies specific sites that may play key functional roles but were obscured by the high natural sequence conservation across the M gene segment. This library also now enables the relation between M1 sequence and function to be investigated by performing selection for traits such as environmental stability, transmissibility, particle morphology, and pH sensitivity.

## MATERIALS AND METHODS

### Generation of the M1 codon mutation library.

Following the published methods of Thyagarajan and Bloom, we used an iterative, low-cycle PCR program with mutagenic synthetic oligonucleotides containing random NNN triplets that overlapped each codon of M1 (70). The PCR template was the M1 coding region of the X31 virus M segment. Three replicate libraries were generated with an average of 2.6 codon mutations per clone. The end primers for the mutagenesis were 5′-CATGATCGTCTCAGGGAGCAAAAGCAGGTAGATATTGAAAG-3′ and 5′-CATGATCGTCTCGTATTAGTAGAAACAAGGTAGTTTTTTACTCCAG-3′, which include BsmBI restriction sites. The final PCR products were gel purified and digested with BsmBI. The M1 library was inserted into dephosphorylated and BsmBI-digested unidirectional reverse genetics vector pHH21 using a T4 DNA ligase (Invitrogen). Column-purified (Zymo Research) DNA plasmids were electroporated into ElectroMAX DH10B T1 phage-resistant competent cells (Invitrogen 12033-015) and plated onto 100 μg/ml of ampicillin LB agar plates. Plating in parallel a 1:4,000 dilution of electroporated cells allowed for counting of individual colonies. We obtained over 1.25 million unique clones per transformation. For each replicate, we performed two transformations to generate about 2.5 million unique clones. Sanger sequencing was performed on 33 miniprepped colonies and analyzed. Transformants from each M1 library replicate were pooled, cultured in LB supplemented with ampicillin, and miniprepped to generate the M1 codon mutant plasmid libraries (Fig. 1). For each replicate, a separate control wild-type M segment in pHH21 vector was created.

### Virus rescue and passage in cells.

The M1 mutant plasmid libraries were used to generate pools of virus using reverse genetics (71). We transfected a coculture of MDCK-SIAT1 cells constitutively expressing TMPRSS2, a transmembrane serine 2 protease needed to activate HA (MDCK-SIAT1-TMPRSS2 cells) (72), and HEK293T cells with equal amounts of the following 9 plasmids: the M1 codon mutant library, the seven other X31 genes in bidirectional reverse genetics plasmids (pHW2000), and a TMPRSS2-expressing plasmid. The use of the constitutively expressed TMPRSS2 in the MDCK-SIAT1 cell line during both the mutant viral reverse genetics rescues and passaging made it possible to eliminate an external protease, such as trypsin, needed to activate HA on newly budding virus particle. Overall, six viral rescues and passages were performed, each using a different M plasmid preparation: the three M1 mutant codon library replicates and three independent unmutated M plasmid replicates.

Each viral rescue was performed by transfecting multiple wells of cells in an effort to increase the diversity of the rescued virus pools and limit bottlenecking. Specifically, 10 wells (2 ml each) were transfected per M1 codon mutant rescue replicate. An additional 2 wells (2 ml each) of control unmutated M plasmid transfections were also included per replicate. For each well, cells were plated at 2 × 10^5^ 293T cells per ml and 2.5 × 10^4^ MDCK-SIAT1-TMPRSS2 cells per ml in 2 ml of D10 (Dulbecco modified Eagle medium [DMEM] supplemented with 10% heat-inactivated fetal bovine serum [FBS], 2 mM l-glutamine, 100 U of penicillin/ml, and 100 μg of streptomycin/ml), and then each well was transfected with 2.25 μg of total DNA (250 ng of each of the nine plasmids) using BioT transfection reagent (Bioland; B01-02). At 12 h posttransfection, the medium was changed to our influenza virus growth media (IGM): Opti-MEM supplemented with 0.01% heat-inactivated FBS, 4% bovine serum albumin (BSA), 100 U of penicillin/ml, 100 μg of streptomycin/ml, and 100 μg of calcium chloride/ml. Viral supernatants were collected and pooled at 72 h posttransfection.

Titers of rescued virus supernatants were determined, and supernatants were passaged at the low MOI of 0.01 through an estimated 1e8 MDCK-SIAT1-TMPRSS2 cells. This low MOI minimizes coinfection early in the passage, thereby ensuring that nonfunctional M1 variants were not propagated and a robust genotype-phenotype link was established. The genotype-phenotype link for each virus in the pool is necessary to ensure that the sequenced M1 gene reflects the M1 protein sequence and is critical for accurate subsequent analysis of gene sequencing (70). Passaged virus supernatants were collected and pooled 40 h postinfection.

### Determination of virus titers.

The titers of the viruses were determined by 50% tissue culture infective dose (TCID_50_). To the first row of a 96-well plate, 10 μl of a 1:10 dilution of the viral supernatant was added to 90 μl of IGM. The virus was then serially diluted 1:10 down the column of the plate. Each plate included 2 samples of no-virus supernatant controls. Fifty microliters of 10^5^ MDCK-SIAT1-TMPRSS2 cells/ml was added to each well. The plates were incubated at 37°C and scored for cytopathic effects 72 h postinfection. The viral titers were calculated by the Reed-Muench analysis implemented by the Python script at https://github.com/jbloom/reedmuenchcalculator.

### Generating and barcoding samples for Illumina deep sequencing.

The deep sequencing samples were prepared by PCR amplicons generated as described previously for the wtDNA, mutDNA, wtvirus, and mutvirus samples, with the exception that we prepared 250-nt paired-end reads for Illumina HiSeq sequencing (70) (Fig. 1). The mutation frequency was determined for the M1 codon library before and after cell selection, yielding the mutDNA (plasmid codon library) and mutvirus (passaged mutant virus) samples in Fig. 1. In order to estimate sequencing error rates, we also deep sequenced wild-type M1 plasmid and passaged virus generated from this plasmid, creating the wtDNA and wtvirus samples in Fig. 1. For wtDNA and mutDNA samples, we directly amplified the M1 gene from the plasmid. For the wtvirus and mutvirus samples, we collected the viral RNA, reverse transcribed the sequence into DNA with M1-specific primers, and then PCR amplified the M1 gene. The viral RNA template was isolated using Qiagen’s RNeasy kit (catalog no. 74104). Reverse transcription was performed with an Accuscript high-fidelity reverse transcription kit (Agilent Technologies) and primers flanking M1 in the pHH21 vector (forward, 5′-AGC AAA AGC AGG TAG ATA TTG AAA G-3′; reverse, 5′-ATT TGC GGC AAT AGT GAG AGG A-3′). Using a DNA standard, we ensured that at least 1e7 copies of cDNA of each sample were further PCR amplified. Through two rounds of PCR, the amplicons were fragmented into 250-nt segments and barcoded using Illunima HiSeq sample barcodes. Sequencing for each replicate consisted of wtDNA, mutDNA, wtvirus, and mutvirus samples (Fig. 1).

To sequence our mutDNA, mutvirus, wtDNA, and wtvirus samples with Illumina HiSeq, we barcoded the M1 gene in 3 subamplicons (250 nucleotides/subamplicon), aiming for ∼1e6 reads/subamplicon with Illumina HiSeq. Most barcoded sequences show a significant number of reads in all samples (Fig. S3A). The exceptions are barcode reads for mutvirus-3 and wtvirus-2 samples, which were lower than expected. However, when we examined our library for the codon counts sampled, we found that there was good coverage of the M1 protein, even for mutvirus-3 and wtvirus-2, observing a coverage of a minimum of 100,000 number of codon counts at every site for all samples (Fig. S3B).

### Analysis of deep sequencing data, inference of amino acid preferences, and raw results.

The deep sequencing data were analyzed with dms_tools2 (https://github.com/jbloomlab/dms_tools2) (46). Computer code that performs all steps in the analysis is available at https://github.com/jbloomlab/Hom_M1_DMS. Note that the repository at https://github.com/jbloomlab/Hom_M1_DMS includes files giving the counts of each codon mutation at each site under each condition, as well as the numerical values of the amino acid preferences across the entire protein.

### Calculation of relative solvent accessible surface area.

Relative solvent accessibility (RSA) based upon a truncated monomeric crystal structure covering residues 2 to 158 (PDB code 1EA3) was calculated with the molecular graphics program Chimera (73, 74) using a probe radius of 1.4 Å and a G-X-G tripeptide as a reference for calculating each amino acid’s fully accessible surface area.

### Data availability.

Deep-sequencing data are available under SRA accession number SRP144482.

## Supplementary Material

Supplemental file 1
