# Supplementary material for: Deep Mutational Scan of the Highly Conserved Influenza A Virus M1 Matrix Protein Reveals Substantial Intrinsic Mutational Tolerance
Source: J Virol. 2019 Jun 14;93(13):e00161-19. doi: 10.1128/JVI.00161-19 (PMC6580950; doi:10.1128/JVI.00161-19)
Supplement: Supplemental file 1 [file JVI.00161-19-s0001.pdf]

Deep mutational scan of the highly conserved influenza A M1 matrix protein reveals substantial intrinsic mutational tolerance

Running title: Deep mutational scan of influenza A M1 matrix protein

Nancy Hom[a], Lauren Gentles[b,c], Jesse D. Bloom[b,c,d], Kelly K. Lee[a,b]#

[a]Department of Medicinal Chemistry, University of Washington, Seattle, WA
[b]Department of Microbiology, University of Washington, Seattle, WA
[c]Division of Basic Sciences, Fred Hutchinson Cancer Research Center, Seattle, WA
[d]Howard Hughes Medical Institute

Address correspondence to: kklee@uw.edu

SUPPLEMENTAL INFORMATION.

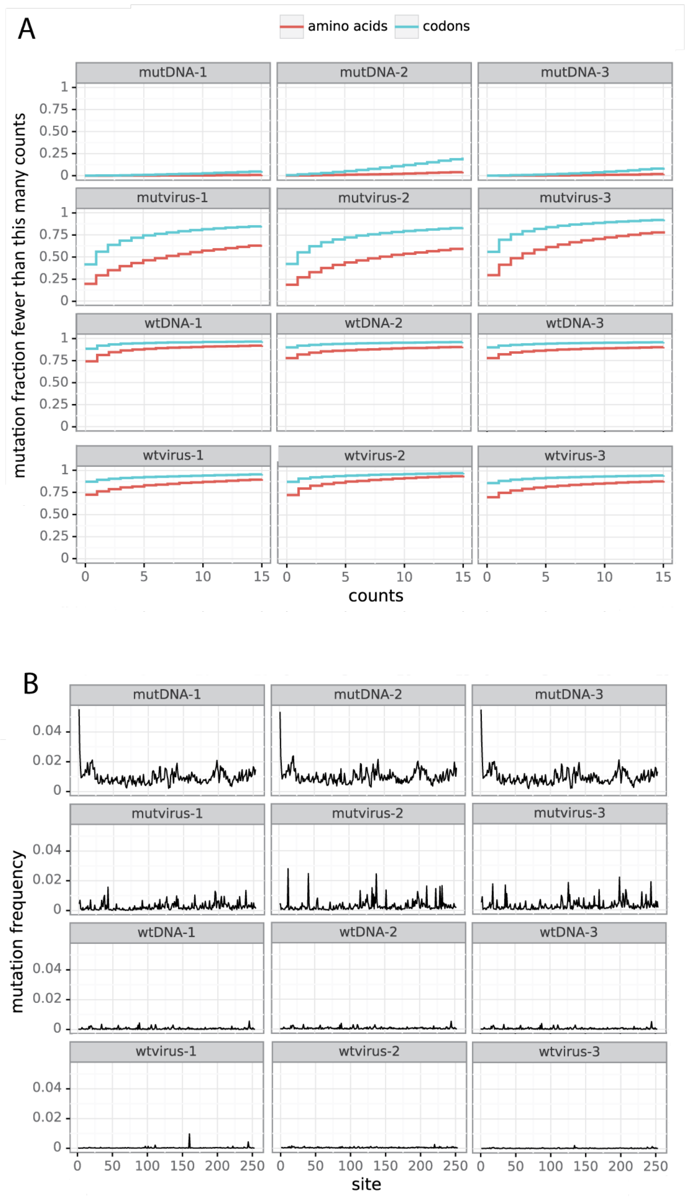

Figure S1. Analysis of mutation sampled in each of the three replicates. (A) Most mutations (~90%) in **mutDNA** have over 15 counts, indicating the initial library thoroughly samples all codon mutations. After replication selection, in the **mutvirus** sample, deleterious mutations are purged, and the fraction of codon mutations with less than 5 counts increases to ~75%. In contrast, most mutations sampled in **wtDNA** and **wtvirus** have low counts; almost 90% of the fractions of mutations have less than 5 counts in both of these

control samples. **(B)** Coverage of mutations across the protein in each of the three replicates demonstrate higher frequency of mutations in the **mutDNA** and **mutvirus** samples than in the **wtDNA** and **wtvirus** samples.

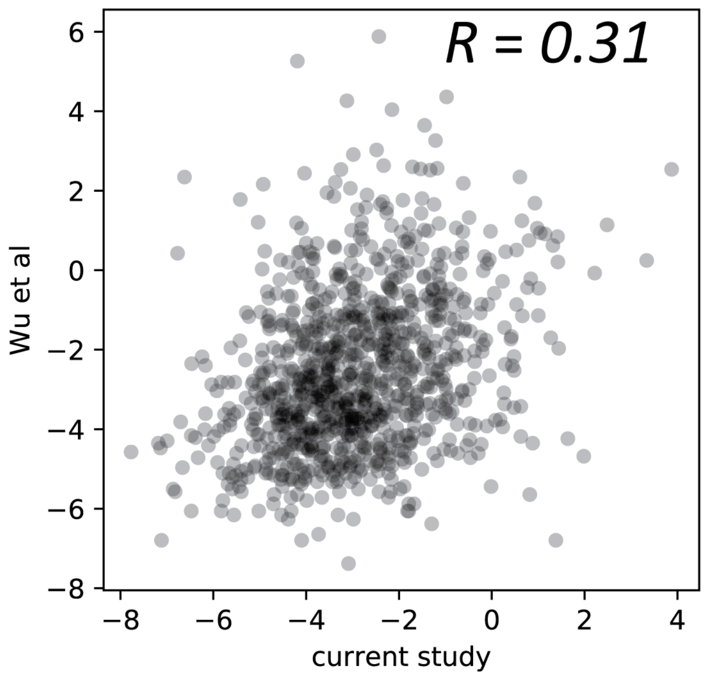

Figure S2. Comparison of DMS analysis to a RT-PCR analysis of M1 amino acid preference by Wu and coworkers (52)**.** A modest correlation as well as clear differences between our results and those from Wu and coworkers is reflected by the relatively low R value of 0.31. We attribute the low correlation to differences in methodology and possibly to differences in influenza virus strain and cell type used for virus passaging.

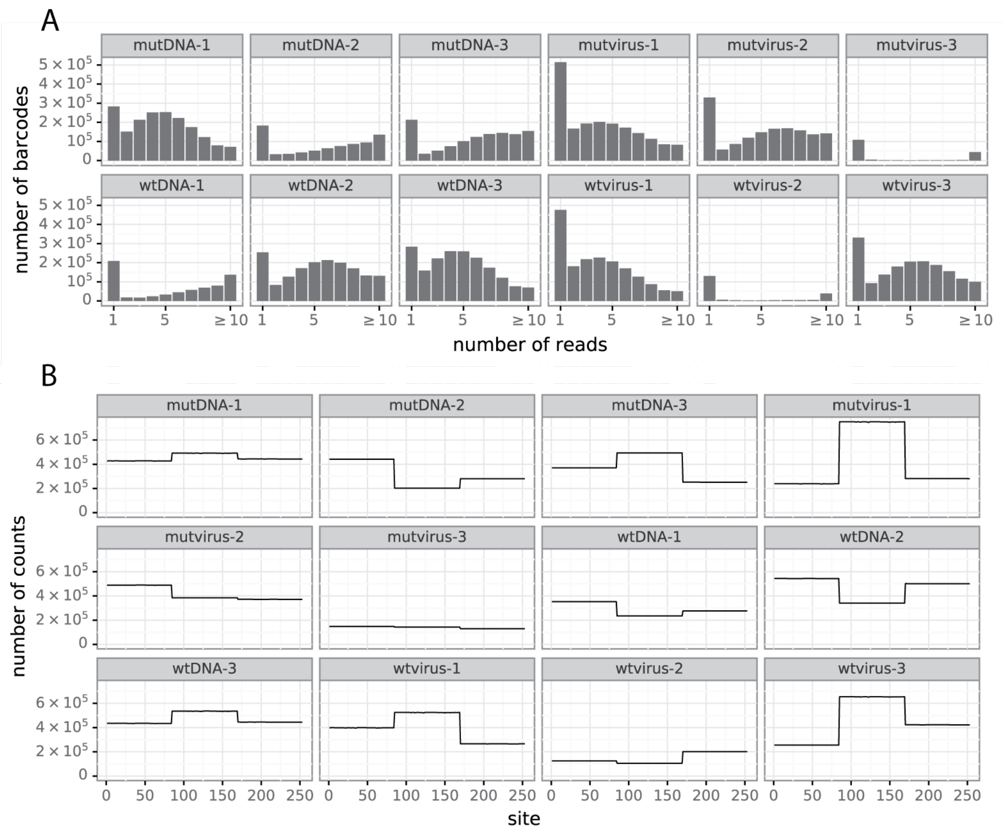

Figure S3. Assessment of the number of Illumina HiSeq barcodes and number of counts. (A) The reads as a function of the number of barcodes indicate there is a significant number of reads in all samples. The only exceptions are in **mutvirus**-3 and **wtvirus**-2 samples. However, there is good coverage of counts across the M1 gene, even in **mutvirus**-3 and **wtvirus**-2 samples, with a minimum of ~100,000 counts per amplicon as plotted in **(B)**.
